# A novel method to quantify local CpG methylation density by regional methylation elongation assay on microarray

**DOI:** 10.1186/1471-2164-9-59

**Published:** 2008-01-31

**Authors:** Dingdong Zhang, Yan Wang, Yunfei Bai, Qinyu Ge, Yingjuan Qiao, Junfeng Luo, Chao Jia, Zuhong Lu

**Affiliations:** 1 State Key Laboratory of Bioelectronics, Southeast University, Nanjing 210096, China; 2 College of Animal Science and Technology, Jinling Institute of Technology, Nanjing 210038, China

## Abstract

**Background:**

DNA methylation based techniques are important tools in both clinical diagnostics and therapeutics. But most of these methods only analyze a few CpG sites in a target region. Indeed, difference of site-specific methylation may also lead to a change of methylation density in many cases, and it has been found that the density of methylation is more important than methylation of single CpG site for gene silencing.

**Results:**

We have developed a novel approach for quantitative analysis of CpG methylation density on the basis of microarray-based hybridization and incorporation of Cy5-dCTP into the Cy3 labeled target DNA by using Taq DNA Polymerase on microarray. The quantification is achieved by measuring Cy5/Cy3 signal ratio which is proportional to methylation density. This methylation-sensitive technique, termed RMEAM (regional methylation elongation assay on microarray), provides several advantages over existing methods used for methylation analysis. It can determine an exact methylation density of the given region, and has potential of high throughput. We demonstrate a use of this method in determining the methylation density of the promoter region of the tumor-related gene *MLH1, TERT *and *MGMT *in colorectal carcinoma patients.

**Conclusion:**

This technique allows for quantitative analysis of regional methylation density, which is the representative of all allelic methylation patterns in the sample. The results show that this technique has the characteristics of simplicity, rapidness, specificity and high-throughput.

## Background

In the human genome, GC-rich DNA sequences are found frequently within the promoter and first exon of ~50% of all genes [1]. These sequences, also known as CpG islands, can be targets of DNA methylation. An epigenetic phenomenon is known to be associated with genomic imprinting and X-chromosome inactivation, and essential for normal mammalian development [2]. Both global hypomethylation and regional hypermethylation have been described in human tumor cell lines and a wide spectrum of cancers [3]. Global hypomethylation has been associated with instability of chromosomal or microsatellite, while regional hypermethylation of CpG islands within promoter region of tumor suppressor genes is associated with transcriptional inactivation and represents an important mechanism of gene silencing in the pathogenesis of neoplasia [4,5]. There is emerging evidence that each tumor may harbor multiple genes susceptible to promoter hypermethylation. Methylation profiles of multiple genes for each cancer type might have important prognostic implications for clinical monitoring, risk assessment, and even therapeutic considerations [6-8].

The present techniques commonly used for the methylation analysis are based on the bisulfite modification of the genomic DNA. Since sodium bisulfite treatment exclusively converts unmethylated cytosine to uracil under appropriate conditions [9], subsequent analysis to differentiate unconverted cytosine from converted uracil enables us to know the primary methylation status. It is the basis for methylation-specific PCR (MSP) [10], bisulfite DNA sequencing [11], enzymatic regional methylation assay (ERMA) [12], pyrosequencing [13], and mass spectrometry [14]. MSP is widely used to analyze promoter methylation, although only a limited number of CpG sites can be analyzed by this method. Bisulfite DNA sequencing provides precise methylation status over an amplified region, but it requires large-scale sequencing of multiple plasmid clones. The major advantage of the pyrosequencing method compared to MSP is that the data are actual sequences rather than fluorescence data from PCR-based amplification. But highly repetitive thymines and the limitation of ~75 bp extension length [15] can affect assay reproducibility and accuracy. Mass spectrometry permits the high-throughput identification of methylation sites and the semiquantitative measurement at single or multiple CpG positions, but this method needs high quality and a large amount of samples, and sometimes false positive results are inevitable. Even though ERMA can determine overall methylation level within a region containing a number of CpG sites, but it requires radioactive labeling of DNA samples and subsequent cumbersome purification steps of the radiolabeled products. Recently, several groups have shown that the methylation status can be achieved by 5mC-antibodies or MBD-proteins [16-18]. However, the affinity and specificity would be further improved for methylation density detection.

Microarrays provide the powerful tools for mapping the epigenome and detecting patterns of DNA methylation in the genome level [19-24]. Methods Coupling CpG-recognizing restriction enzymes with microarray technique have been reported, such as differential methylation hybridization (DMH) [19,23], methylation amplification DNA chip (MAD) [22], and microarray-based integrated analysis of methylation by isoschizomers (MIAMI) [21]. The above methods are only able to analyze methylation at the restriction sites of the enzymes, which are potentially biased by mutations or polymorphisms at the sites. Another microarray approach for methylation pattern analysis is methylation-specific oligonucleotide (MSO) microarray [19,20]. MSO uses bisulfite-treated DNA as template for non-discriminatory PCR amplification, followed by hybridization of the PCR products to glass slides with oligonucleotides that could discriminate between methylated and unmethylated cytosines at specific CpG positions. One of the great potentials of MSO is that multiple genes can be analyzed on the same array. A potential limitation of this method is that closely spaced CpGs may not be amenable to analyze if the gene in question is heterogeneously methylated. Recently, genomic tiling microarray has been used to profile DNA methylation patterns [24]. But this method can not obtain exact informations of methylation level within the given region of specific caner-related genes.

Here, we describe a novel technique for the quantification of CpG methylation density of a given DNA region, which combines microarray-based hybridization and enzymatic elongation on microarray. The quantification is achieved by measuring Cy5/Cy3 signal ratio which is proportional to methylation density. In contrast to the other protocols, this method determines the methylation density of the entire amplified region, not only of a few CpG sites or those CpG dinucleotides that are covered by PCR primers/probes. The results show that this technique allows for the quantitative analysis of regional methylation density with simplicity, rapidness, specificity and high-throughput.

## Results

### Principle of the method

**Figure 1:**
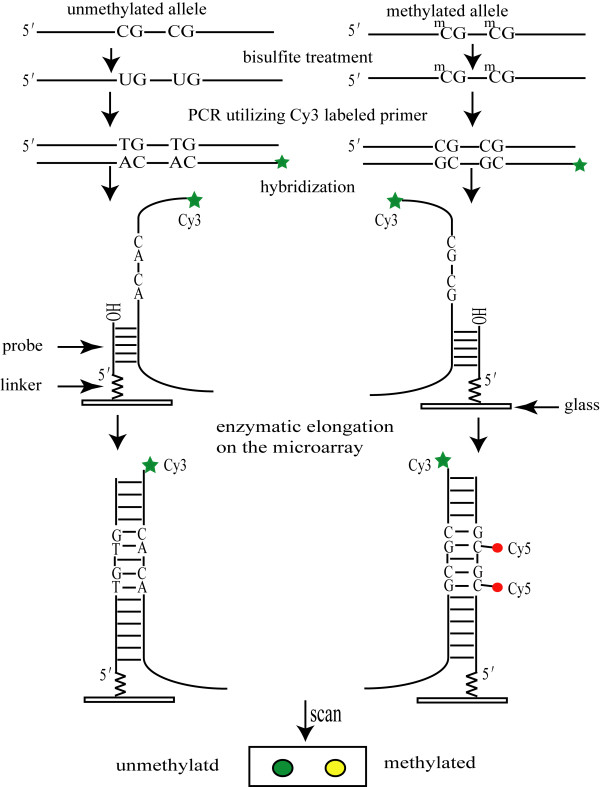
Outline for the analysis of gene promoter methylation density.

Figure 1 outlines the RMEAM (regional methylation elongation assay on microarray) strategy for DNA methylation density analysis. Test DNA samples are bisulfite-modified, PCR amplified products which contain pools of DNA fragments with altered nucleotide sequences due to their differential methylation status. As shown, the unmethylated allele of a given DNA sequence is expected to have the unmethylated cytosine of the test CpG sites converted to thymine, whereas these CpG sequences remain unchanged in the methylated allele. The information on cytosine methylation can now be converted into sequence information. The region of interest is amplified with primers that are specific for bisulfite-modified DNA without CpG dinucleotides in the sequence, which ensures that the PCR amplification is independent of the original methylation status. Thus, the number of CpG dinucleotides remaining in the amplified region would reflect original methylation status. Subsequently, the forward primer was modified with -NH_2 _at 5' end, and then fixed on the aldehyde coating glass as probe leaving 3'-OH free. Target DNA is then hybridized to arrayed oligonucleotide probes which are specifically designed to match the reverse strands. For quantification of CpG dinucleotides, enzymatic elongation was carried out with dNTPs (dATP, dGTP, dTTP, dCTP, Cy5-dCTP), PCR buffer, Mg^2+ ^and Taq enzyme on microarray. The incorporation of Cy5-dCTP into the DNA is proportional to the number of methylated CpG sites originally present in this DNA region. To minimize the background signal and solve the problem that a variable quantity of DNA has different fluorescence intensities in every reaction tube, we devised a smart way to accurately standardize between samples. For this step, we modified Cy3 at the 5'end of reverse primers and the Cy3 labeled reverse strands were hybridized to the oligonucleotide probes. Because the number of Cy3 is identical for every PCR product, the Cy3 signal can be used as an internal control to standardize the amount of DNA that is finally analyzed. The results are expressed as fluorescence intensity ratio (Cy5/Cy3). By use of the mixture of fully-methylated and non-methylated alleles as standards, the ratios can be converted into percentage values, and thus, the average methylation density of the amplified region is determined. However, considering standardization of Cy3 and Cy5 fluorescence intensity as well as quality control, we use a positive control (fully-methylated APC allele from clone) just like the housekeeping gene of cDNA microarray and a negative control which removes the influence of cross-hybridization. This positive control has definite number of CpG sites and merges the PCR products before the hybridization. Thus, the fluorescence intensity ratios (Cy5sam*Cy3pos)/(Cy5pos*Cy3sam) will be proportional to the methylation density in the original sequence (sam: sample; pos: positive control).

### Optimization of assay conditions for the *MLH1*, *TERT *and *MGMT *promoter region

**Figure 2:**
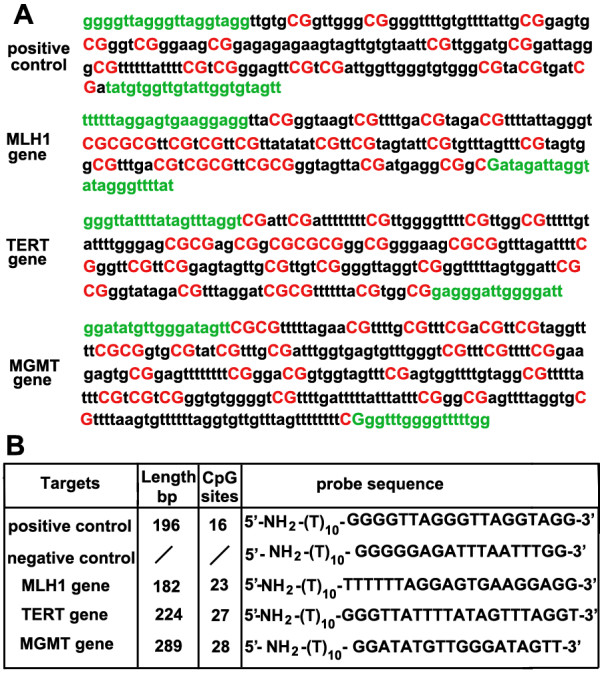
**A**: Fully-methylated sequences of the 5' untranslated region of the positive control (fully-methylated APC allele from clone), *MLH1*, *TERT *and *MGMT *gene are displayed. The strand shown was amplified using the primers (green color) which are specific for bisulfite-modified DNA without CpG dinucleotides. **B**: The nucleotide sequences of probes, the length of targets and the number of CpG sites are shown.

**Figure 3:**
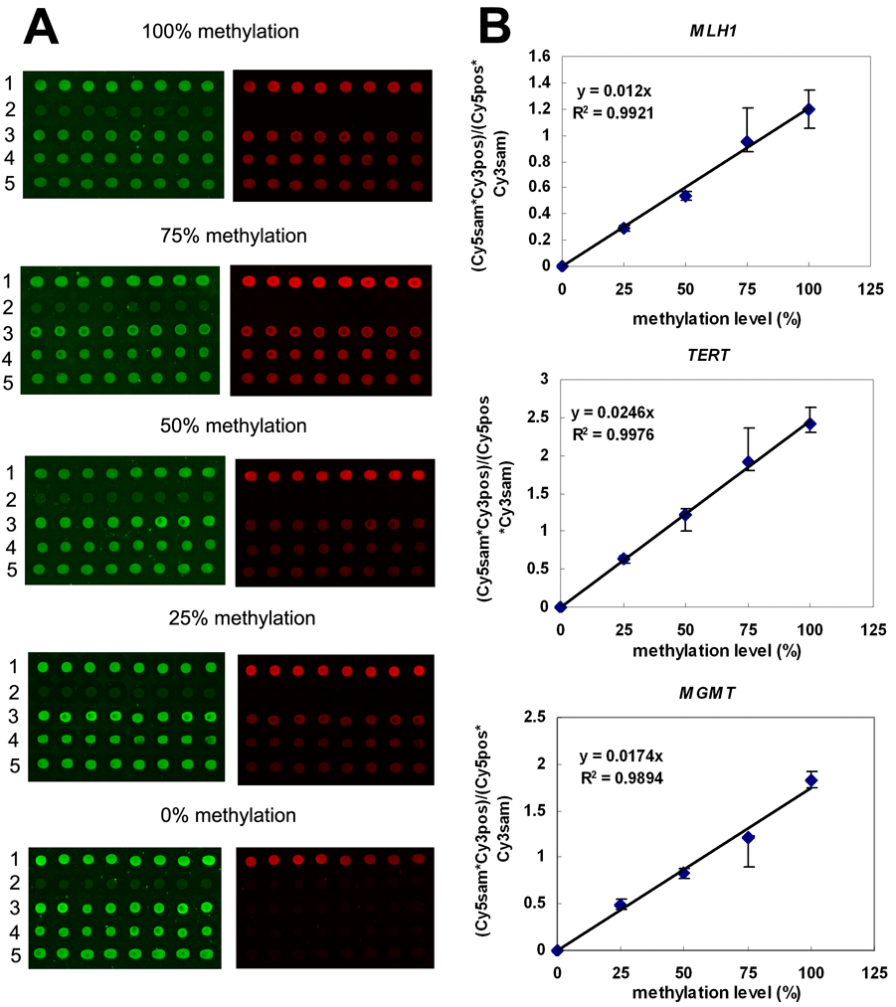
**A**: Fully methylated and unmethylated DNA were mixed (0%, 25%, 50%, 75%, 100%) and hybridized to microarray. The Cy3 green and Cy5 red fluorescence signals of *MLH1*, *TERT *and *MGMT *promoter region are shown, reflecting the indicated percentage of methylation. From top to bottom, the five probes are positive control (1), negative (2), *MLH1 *(3), *TERT *(4) and *MGMT *(5). **B**: Three calibration curves for measuring methylation densities in *MLH1*, *TERT *and *MGMT *promoter region. The intensity ratios (Y-axis) represent signal intensities of (Cy5sam*Cy3pos)/(Cy5pos*Cy3sam).

We determined the feasibility of this RMEAM strategy by assessing the methylation density of CpG islands located in the promoter regions of *MLH1, TERT *and *MGMT *(Fig. 2A), and it has been proved that the mathylation of *MLH1*, *TERT *and *MGMT *are associated with carcinogenesis of colorectal tumor. A group of forty-arrayed oligonucleotides (Five rows of probes which have eight replicated spots; See Fig. 2B and Fig. 3A) were designed to hybridize with the labeled PCR products. The number of CpG sites in positive control (APC), *MLH1, TERT *and *MGMT *were 16, 23, 27 and 28 respectively (Fig. 2A). Fully-methylated and non-methylated DNA targets amplified from clones of these three genes were used to test the accuracy and reproducibility of the RMEAM method. A 182-bp fragment of the *MLH1 *promoter region, a 224-bp fragment of the *TERT *promoter region and a 289-bp fragment of the *MGMT *promoter region were amplified by triplex PCR using primers that were specific for bisulfite-converted DNAs (Fig. 2B). The fully-methylated alleles generated in this way had 100% unconverted cytosine in the test CpG sites, whereas the non-methylated alleles had all cytosine residues converted into thymine in the amplified DNA.

To evaluate the sensitivity and quantitative accuracy of our assay as well as determining any possible PCR bias [25], we then carried out mixing experiments using methylation positive clones and methylation negative clones. The mixtures were prepared prior to PCR in order to assess the possibility of bias during the PCR amplification [12]. The mixtures of Cy3-labeled fully-methylated allele and non-methylated allele with different proportion merged the Cy3-labeled positive control. And then a series of microarray hybridization were performed. After hybridization, elongation was carried out. Optimal elongation conditions on microarray were determined in order to allow elongation reactions to go to completion. Figure 3 shows that there is a linear correlation between the defined methylation density of the standard clone dilutions and the (Cy5sam*Cy3pos)/(Cy5pos*Cy3sam) signal ratios. By including mixtures of clones as standards (fully-methylated only, 75% fully-methylated/25% non-methylated, 50% fully-methylated/50% non-methylated, 25% fully-methylated/75% non-methylated and non-methylated only, corresponding to 100%, 75%, 50%, 25% and 0% methylation density) in every assay [12], it is possible to create a standard curve in order to control for differential specific activity and incorporation efficiency of the Cy5-dCTP. This standard curve can then be used to calculate the methylation density of unknown samples from the ratios (Cy5sam*Cy3pos)/(Cy5pos*Cy3sam). All standards and unknown samples were analyzed with eight replications.

### Verification of RMEAM findings by bisulfite sequencing

**Figure 4:**
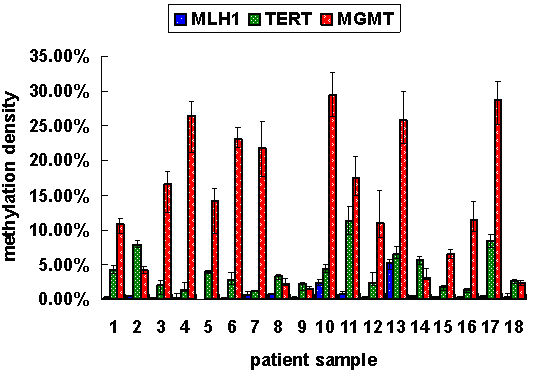
*MLH1, TERT *and *MGMT *promoter methylation densities of tumor samples from 18 patients with colorectal tumor.

In order to validate the accuracy of the RMEAM approach, we analyzed the *MLH1, TERT *and *MGMT *promoter methylation density in 18 colorectal carcinoma patients. Figure 4 showed that this assay could detect precisely the methylation density in every patient sample. In these samples, *MLH1 *promoter had 0%–5.21% methylation density (mean, 0.78%); *TERT *promoter had 1.18%–12.59% methylation density (mean, 4.48%) and *MGMT *promoter had 1.55%–30.84% methylation density (mean 14.44%). The region of *MLH1 *promoter in this assay was selected according to Maekawa et al [26]. This region is a little farther than the region relative to the transcriptional start site of *MLH1 *described by Deng et al. [27]. Consequently the methylation level of this region is relative low.

**Figure 5:**
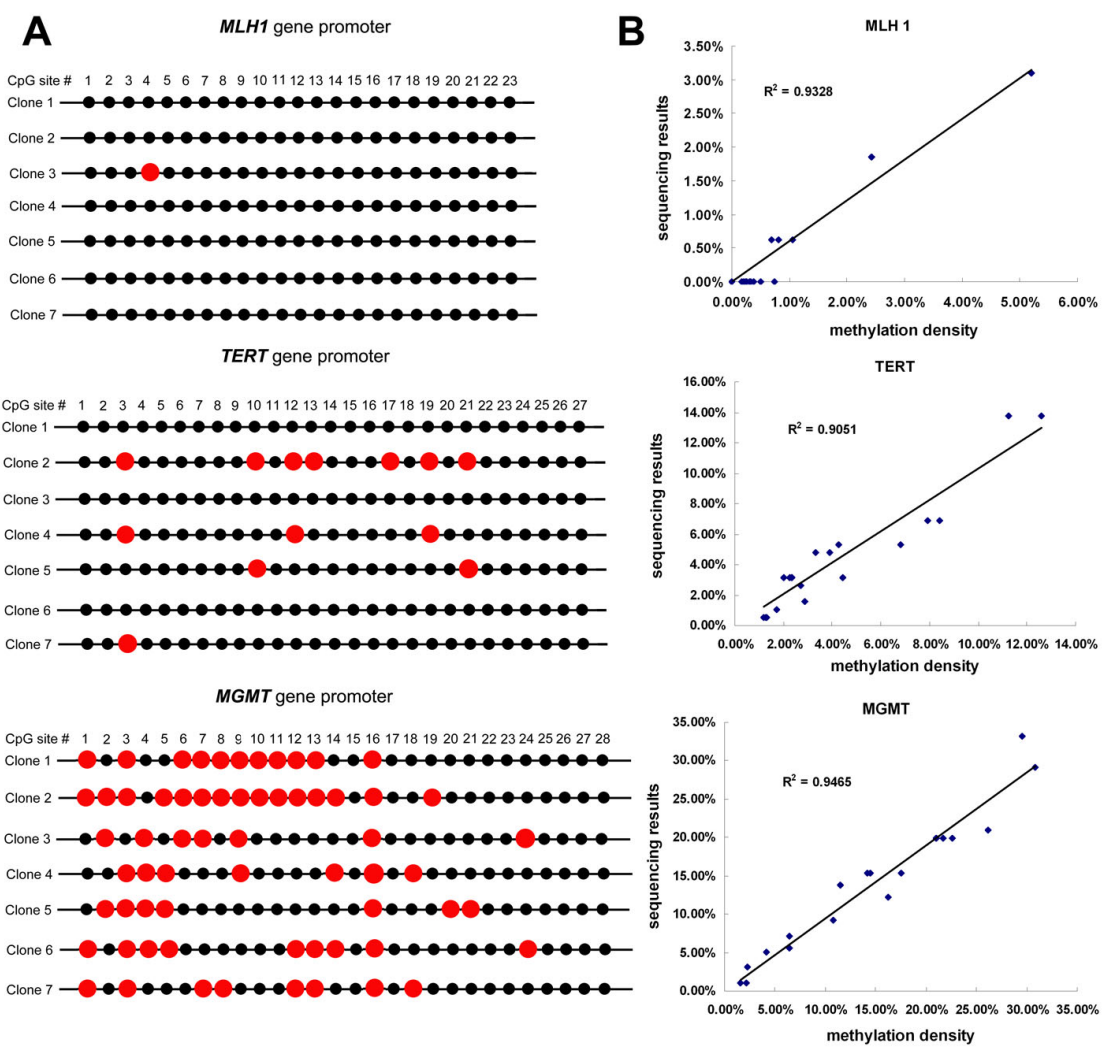
**A**: Representative sequencing data of patient No.17 was shown. Red circle indicates the methylated CpG site. 7 clones or less per gene in each patient sample were sequenced. **B**: Correlations between bisulfite sequencing results and methylation density data of the *MLH1, TERT *and *MGMT *promoter region determined by our new assay in 18 patients with colorectal carcinoma.

When we compared our RMEAM results with succedent bisulfite sequencing data, a high concordance between both methods was found. Figure 5 showed the correlations between the results obtained by RMEAM with the data from bisulfite sequencing in *MLH1, TERT *and *MGMT *promoter regions of 18 colorectal tumor patients.

## Discussions

We developed a novel approach for quantitative analysis of CpG methylation density on the basis of microarray-based hybridization and incorporation of Cy5-dCTP into the Cy3 labeled target DNAs by using Taq DNA Polymerase on microarray. This method provides several advantages over existing methods for quantitative methylation analysis. Firstly, it has potential for high-throughput analysis of DNA methylation profiles. CpG island hypermethylation has been reported to be linked to the silencing of many cancer-related genes. A DNA microarray can be designed and generated to contain a large amount of oligonucleotide probes capturing specifically target genes. Cancer-related genes can be parallelly amplified from investigated samples in a 96-well format or with multiple PCR, which can generate multiple target genes for hybridization and detection. Secondly, RMEAM can determine the methylation density of all CpG dinucleotides within the entire amplified region other than individual CpG sites. In the case of the *MGMT *promoter region, the detected methylation density includes the methylation status of 28 CpG dinucleotides. RMEAM produced a linear response when used for quantitative methylation analysis in mixing experiments by use of DNA from fully-methylated and non-methylated clones with a defined methylation status. The third feature of RMEAM is its simplicity and precision. Unlike ERMA method [12], RMEAM doesn't require radioactive labeling of DNA samples and subsequent cumbersome purification steps of the radiolabeled products in tubes. Hybridization, elongation and washing on microarray can be quickly and easily achieved within 4 hours. The use of an internal control (Cy3) allows for correction of potential substrate loss and for normalization of the results to the DNA amount that is finally analyzed by microarray scaner. The negative control in Figure 3 can remove the influence of cross-hybridization. For example, the net Cy3 intensities of row 1, 3, 4 and 5 of Figure 3A can be derived by subtraction of the row 2 negative control background. In row 2, the non-specific hybridization can't bring incorporation of Cy5-dCTP into the DNA due to the specificity of Taq DNA Polymerase. Dual-color fluorescence can be standardized by positive control, just like house-keeping gene in cDNA microarray. The accuracy of our new technique is shown by the high concordance with bisulfite sequencing data. However, in this study, only 7 clones or less per gene in each patient sample were sequenced, which might account for the slight discrepancies in methylation percentages (Figure 5). As cloning and final sequencing of a small number of alleles does not necessarily give representative data for the initial sample, the approach used in our current assay can provide more informations regarding the overall methylation density of the region from the sum of all alleles. Furthermore, RMEAM approach is less costly and labor intensive than the extensive bisulfite sequencing analysis needed for precise quantitation.

One possible limitation of our method is that it is not suitable for a detailed characterization of the pattern of DNA or the determination of methylation status of individual CpG sites. However, since difference of site-specific methylation may also lead to a change of methylation density in many tumor-related genes, It has been indicated that the methylation density of promoter CpG islands is more important than methylation occurred at a specific single CpG site for gene silencing [28-32]. Nevertheless, it has been very difficult to measure the level of methylation in a target area containing multiple CpG sites. Results with our new assay might be more biologically relevant, especially in patient samples with heterogeneous methylation patterns. Furthermore, it was suggested in an in vitro model that de novo CpG island methylation is not a single event, but rather a progressive process, and appears to be region specific [33]. Therefore, it is reasonable to apply a method that accurately determines the methylation density of a CpG island region in order to investigate the dynamics of methylation changes in human malignancies.

One of the potential applications of RMEAM approach is to examine the changes in methylation patterns following treatment with demethylating drugs [34]. Galm et al. [12] had demonstrated the application of ERMA technique for the quantitative analysis of methylaiton density, in which KG1a cells were treated in vitro with the increasing 5'aza-2'-deoxycytidine (DAC) dose within a given time course. The findings in that paper indicated that the changes of methylation density could help to optimize the dosage of demethylating drugs for clinical use by determining the extent of demethylation at a dose range with little cytotoxic side effects. Large-scale screenings of tumor-related genes under various conditions are needed to elucidate the functional role of DNA methylation and to screen for new antineoplastic drugs operative on that mechanism. Our new method could help elucidate the consequences of treating patients with such drugs and eventually help clarify their therapeutic value in future antineoplastic therapy regimens. Recent evidence has indicated that increased density of methylation within a susceptible CpG island is associated with more advanced stages of tumors. Our RMEAM method coupled with quantitative real-time PCR approaches to detect gene expression levels will contribute to more accurately assess the dynamics of methylation-mediated transcriptional silencing of tumor-related genes in carcinogenesis. Such assays would preferably be genome-wide to monitor both desired and undesired effects on methylation and gene expression.

## Conclusion

We described a novel approach for quantitative analysis of CpG methylation density on the basis of microarray-based hybridization and incorporation of Cy5-dCTP into the Cy3 labeled target DNA by using Taq DNA Polymerase on microarray. Our results showed that this RMEAM technique allows for the quantitative analysis of regional methylation density with simplicity, rapidness, specificity and high-throughput.

## Methods

### Patients and DNA extraction

Informed written consent was obtained from all patients and donors, and tissue collection was approved by each Institutional Review Board. The samples of primary tumor tissues were collected from colorectal carcinoma patients (n = 18) during surgery at Gulou Hospital and Zhongda Hospital (Nanjing, China). All tissue samples were fresh-frozen and stored at -80°C until further processing. Genomic DNA was extracted from tissues by digesting with proteinase K (0.5 mg/ml) in 10 mM TE (pH 8.0) buffer and 0.5% SDS at 48°C overnight, followed by a standard phenol/chloroform (1:1) extraction and precipitated with ethanol in a standard fashion.

### Bisulfite modification of genomic DNA

Bisulfite treatment was carried out following the modified [35] procedure of Frommer et al. [11]. Briefly, 5 μg of genomic DNA were digested with EcoRI (New England Biolabs) and denatured with 0.3 M NaOH for 15 min at 37°C. A freshly prepared solution of sodium bisulfite (2.5 M, pH 5.0) and hydroquinone (100 mM) was added to the denatured DNA, and the mixture was incubated at 55°C for 5 h. After desalting (Wizard Clean Up System; Promega), the DNA was desulphonated with 0.3 M NaOH for 15 min at 37°C. The solution was neutralized with 75 μl of 5 M ammonium acetate (pH 7.0), and the DNA was ethanol precipitated and resuspended in Tris-EDTA (pH 7.5).

### Preparation of the positive and negative clones

Placental DNA treated *in vitro *with *Sss*I methyltransferase (New England Biolabs) was used as fully-methylated alleles for *APC*, *MLH1, TERT *and *MGMT *gene. Meanwhile, DNA from normal lymphocytes was used as unmethylated alleles for *MLH1, TERT *and *MGMT *gene. Sodium bisulfite modification of the unmethylated DNA and M. SssI-treated DNA were then performed. The targeted CpG islands (see Fig. 2) were amplified from bisulfite-treated genomic DNA by triplex PCR using the following primer pairs [26,36,37]. The primers used were *MLH1*-Forward, 5'-TTTTTTAGGAGTGAAGGAGG-3'; *MLH1*-Reverse, 5'-ATAAAACCCTATACCTAATCTATC-3'; *MGMT*-Forword, 5'-GGATATGTTGGGATAGTT-3'; *MGMT*-Reverse, 5'-CCAAAAACCCCAAACCC-3';*TERT*-Forward, 5'-GGGTTATTTTATAGTTTAGGT-3'; *TERT*-Reverse, 5'-AATCCCCAATCCCTC-3'. These primers are specific for bisulfite-modified DNA without CpG dinucleotides. The PCRs were performed in 25-μl reactions containing PCR buffer with 1.8 mM MgCl_2_, 5 pmol primer, 1.25 units Hot-start DNA polymerase (TaKaRa), and 1 μl bisulfite-modified DNA (equivalent to 50 ng genomic DNA). After an initial preheating step of 5 min at 95°C, PCR was performed 40 cycles of 95°C for 30 s, 54°C for 30 s, and 72°C for 30 s, with a final extension at 72°C for 7 min. APC fully-methylated allele was used as positive control which was amplified by forward primer 5'-GGGGTTAGGGTTAGGTAGG-3' and reverse primer 5'-AACTACACCAATACAACCACATA-3' [36]. All PCR products were gel purified and cloned into the pMD18-T vector according to the manufacturer's instructions (TaKaRa) and sequenced using the ABI sequencing system. Quantitation of the PCR product was performed with a UV spectrometer; 1 OD_260 _unit was calculated as 50 ng/μL.

### Fabrication and hybridization of microarray

Five probes (18–20 nucleotides in length, Fig. 2) were synthesized with an amino-linked C6 [NH_2 _(CH_2_)_6_] linker attached to its 5'end. Additional 10 nucleotides T were used to reduce the space influence. Each oligonucleotide was printed on the aldehyde-coated glass slides using a PixSys5500 microarrayer (Cartesian Technology Inc). After printed, the glass slides were incubated in a humid chamber at room temperature overnight, and then at 37°C for 2 h. The slides were washed thoroughly in 1% SDS solution to remove unbound oligonucleotides. After further treatment with a NaBH_4 _solution for 20 min, the slides were ready for hybridization. For target DNA labeling, PCR products of bisulfite-treated DNA were labeled at the 5'end of reverse primer with Cy3. The labeled products were resuspended in hybridization solution (1:3 dilution v/v). Then the mixture was denatured at 95°C for 5 min, immediately cooled on ice for 10 min and subsequently applied to the DNA microarray slides. Microarray hybridization was conducted in a moist hybridization chamber under a cover slip at 42°C for 3 h recommended by Schumacher et al [38].

### Elongation on microarray

After hybridization, the slide was rinsed and washed at room temperature with 2 × SSC-0.1% SDS for 10 min, 0.1 × SSC-0.1% SDS for 5 min, water for 5 min, and then dried by flowing nitrogen. Then 15 μL elongation system was applied to the hybridization region with a cover slip. The elongation system contained 1 × PCR buffer, 2.5 mM Mg^2+^, dNTPs (20 μM dGTP, 20 μM dATP, 20 μM dTTP, 0.2 μM dCTP, 0.2 μM Cy5-dCTP), and 1.5 IU Taq DNA Polymerase (TaKaRa). The incubation procedure was 42°C 10 min, 55°C 5 min, and 72°C 3 min. Subsequently, the slide was washed with 2 × SSC-0.5% SDS and dried, and it was ready to be scanned.

### Image scanning and data processing

These microarray slides were scanned with ScanArray Lite microarray analysis systems (A Packard BioScience Company, USA). The fluorescence images were analyzed with GenePix Pro3.0 software. Each spot was defined by the positioning of a grid of circles over the array image. For each fluorescent image, the average pixel intensity within each circle was determined and a local background using mean pixel intensity was computed for each spot. Net signal was determined by subtraction of this local background from the mean average intensity for each spot and the nospecific influence of cross-hybridization from negative control. Signal intensities of individual spots were obtained and exported to Excel spreadsheets for further analysis. The intensity ratio of Cy5/Cy3 for each probe set was then obtained.

## Authors' contributions

DZ and ZL designed the study and wrote the manuscript, YW performed the fabrication of microarray and microarray hybridization, YB and QG provided the positive and negative clones of *APC*, *MGMT*, *MLH1*, *TERT *genes, YQ performed bisulfite modification and PCR, JL provided genomic DNA of colorectal cancer tissue samples, and CJ performed data analysis. All authors read and approved the final manuscript.
